# Electronic structure and reactivity of tirapazamine as a radiosensitizer

**DOI:** 10.1007/s00894-021-04771-8

**Published:** 2021-05-22

**Authors:** José Romero, Thana Maihom, Paulo Limão-Vieira, Michael Probst

**Affiliations:** 1 Institute of Ion Physics and Applied Physics, University of Innsbruck, Technikerstraße 25, 6020 Innsbruck, Austria; 2 Atomic and Molecular Collisions Laboratory, CEFITEC, Department of Physics, Universidade NOVA de Lisboa, 2829-516 Caparica, Portugal; 3 School of Molecular Science and Engineering, Vidyasirimedhi Institute of Science and Technology, Rayong, 21210 Thailand

**Keywords:** Tirapazamine, Radiosensitizer, Radiation, Cancer, Hypoxia, Density functional theory

## Abstract

Tirapazamine (TP) has been shown to enhance the cytotoxic effects of ionizing radiation in hypoxic cells, thus making it a candidate for a radiosensitizer. This selective behavior is often directly linked to the abundance of O_2_. In this paper, we study the electronic properties of TP in vacuum, micro-hydrated from one up to three molecules of water and embedded in a continuum of water. We discuss electron affinities, charge distribution, and bond dissociation energies of TP, and find that these properties do not change significantly upon hydration. In agreement with its large electron affinity, and bond breaking triggered by electron attachment requires energies higher than 2.5 eV, ruling out the direct formation of bioactive TP radicals. Our results suggest, therefore, that the selective behavior of TP cannot be explained by a one-electron reduction from a neighboring O_2_ molecule. Alternatively, we propose that TP’s hypoxic selectivity could be a consequence of O_2_ scavenging hydrogen radicals.

**Supplementary Information:**

The online version contains supplementary material available at 10.1007/s00894-021-04771-8.

## Introduction

According to the World Health Organization, cancer is one of the leading causes of death [1]. In 2017, the USA alone reported cancer to be the second most probable reason of death, only surpassed by cardiovascular diseases [2]. *Hypoxia* is a common trait in solid tumors [3], known to decrease the efficiency of radiotherapy [3–6], since O_2_ is a strong radiosensitizer [7]. On average, ‘normoxic’ tissue in mammals consists of 2 to 9% of O_2_, anything below 2% is classified as hypoxia, while below 0.02% as severe, or acute, hypoxia [7].

The Oxygen Fixation Hypothesis (OFH) is the most widely accepted theory to explain how O_2_ could induce permanent DNA damage [3, 4, 7, 8]. It proposes that the incoming ionizing radiation (IR) does not damage the DNA structure directly as it penetrates the cells, but does so instead indirectly by producing reactive oxygen species (ROS) [4, 7, 8]. On the other hand, direct interaction of IR with DNA forms ion pairs in its structure, which are unaffected by the neighboring O_2_ molecules [9].

A particular ROS of biological interest are hydroxy radicals, , as they are capable of reacting with all four bases of DNA. By doing so, an intermediate radical is produced, which then reacts with a neighboring O_2_ molecule [11], potentially causing a more severe and permanent DNA lesion, given that they are harder to repair [7, 9]. If no O_2_ is available, however, these free radicals, in the DNA structure, are neutralized by radical scavengers, such as glutathione (GSH). As a by-product of GSH reacting with other radicals, glutathione disulfide (GSSG) is produced [9]. Thus, reduced ratios between GSH and GSSG are indicators of poor cellular health [12].

The diminished radiosensitizing effect is often compensated by using radiosensitizing molecules. These molecules serve as a replacement of O_2_ in the chemical reactions that potentially lead to DNA damage [6], since O_2_ is a powerful radiosensitizer. Ideally, a good radiosensitizing molecule should not be metabolized by the cells, in order to enhance its diffusion in hypoxic environments [13], such as tumors. Using these molecules, one can potentially decrease the chances of a patient developing a secondary cancer from radiotherapy, as lower radiation doses might be required to achieve the same desired effects. However, one must always consider the possible toxicity and/or side effect these molecules may have on a patient in high concentrations.

### Tirapazamine

Tirapazamine (TP) has been proposed as radiosensitizer candidate due to its unique targeted cytotoxic mechanism to hypoxic environments [14, 15]. Dorie and Brown (1993) reported an enhancement of tumor cell killing, both in vitro and in vivo, when TP was administered 2 to 3 hours before adding the platinum-based chemotherapy drug cDDP, known as cisplatin [16]. Siim et al. (1997) also demonstrated that TP potentiates cell death when fractioned radiation is administered [17]. Moreover, in a phase II clinical trial of TP combined with cisplatin conducted by Bedikian et al. (1997), a higher activity was detected, compared to cisplatin alone, with only mild side effects when administered intravenously [18].

TP’s selective cytotoxicity has been linked to the formation of a DNA damaging radical which is back oxidized to its parent neutral state if O_2_ is present [10, 14]. The formation of this TP radical is further supported by a study by Brown (1990) [19], which concluded that dimethyl sulfoxide (DMSO), added simultaneously with TP, leads to an increased survival rate of the cells.

**Fig. 1:**
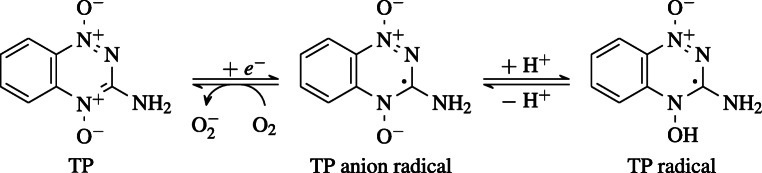
Proposed deactivation and radical formation mechanisms of TP. When exposed to oxygen, a TP anion is neutralized, becoming unable to form a DNA damaging radical species [10]

In its anion state, TP is a radical, capable of forming two DNA damaging species, either a hydroxyl or a benzotriazinyl (BTZ) radical, when protonated [10]. Another study by Siim et al. (2004) showed that coadministering TP with SR 4317 at high, but nontoxic, levels potentiated its cytotoxicity [20]. SR 4317 is a molecule similar to TP, with its sole difference being the absence of OH from the rightmost radical of Fig. 1.

### Calculations

**Fig. 2:**
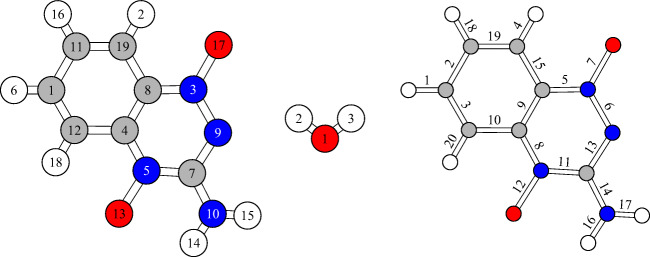
Structure of TP and water molecules, along with the numbers assigned to each of their atoms, and the numbers assigned to the bonds of TP

Very few computational studies on TP have been performed to date. Ban et al. (2001) showed how TP reacts with a DNA sugar-$\text {C}_{1}^{\prime }$ radical and proposed several mechanisms, using Density Functional Theory (DFT) calculations [21]. They concluded that TP must be protonated in order to oxidize the sugar-$\text {C}_{1}^{\prime }$ radical. They also found that the preferred reaction mechanism is a direct attack of one of the oxygens of TP that are bound to N, followed by cleavage of the N-O bond (bond 12, Fig. 2).

Recently, Arthur-Baidoo et al. performed experimental studies accompanied by DFT calculations on the low-energy electron attachment to TP in the gas phase [22]. They predominantly observed the formation of TP anion fragments missing both an oxygen and a hydrogen. Based on DFT calculations, they proposed an OH roaming mechanism competing with OH dissociation to explain the experimentally observed reaction channels.

In this work, we investigate some of the properties of TP and its reactions that are relevant for a potential application as a radiosensitizer by means of computational quantum chemistry. We investigate TP in vacuum but take special care to include the effects of surrounding water molecules. For that, we investigate the effects of micro-hydration and model hydration with a Continuum Model (PCM). We take special care to find realistic positions of the surrounding water molecules by global optimization methods. We analyze electron affinities and electron distribution differences between TP and its anion, both isolated and hydrated. We also discuss the effect of water molecules on the electronic density distribution of TP. We analyze the free energies of the most accessible bond dissociation reactions of both neutral TP and its anion and present energy profiles for some of these reactions.

We assess the accuracy of our DFT calculations, namely for the H-bonding anionic systems, using several density functionals. While GD3-B3LYP is a widely popular functional, it is known to have several deficiencies concerning hydrogen-bonded systems. The functional M06-2X [23] strives to overcome this issue by including dispersion interaction in the functional itself [24]. Improvements of the B3LYP functional have also been made, like for example in the newer CAM-B3LYP functional [25]. Finally, we also include comparisons with the functional *ω* B97XD [26] which accounts for long-range corrections by adding empirical atom-atom dispersion corrections in the functional.

## Methods

Electron densities, electron affinities, and the energetics of the decay reactions were derived from quantum chemical calculations. For solvated TP, we employ a PCM [27–29] which embeds TP in a polarizable medium with the dielectric constant of water. Investigating the effects of micro-hydration requires knowledge of all representative TP/water conformations. This is not an easy task due to the multiple minima of the energy hypersurface caused by various competing binding sites. We adapt the following strategy: An initial geometry optimization, using the MMFF94 force field [30], is followed by geometry optimization with the B3LYP [31, 32] hybrid functional and the cc-pVDZ basis set [33], including the empirical dispersion “GD3” [34].

While this approach leads to “reasonable” conformations which can, however, still be far from the global energy minimum, only local optimization algorithms are applied, and hence used exclusively to determine the geometry of an isolated TP molecule. Therefore, when attaching molecules of water to TP, we add an extra optimization step, where we treat both TP and all molecules of water as rigid objects, thus reducing the number of optimization variables. All water-water interactions are modeled with the TIP3P pair potential [35–37], and all TP-water interactions through Jorgensen’s CM1A potential function [38], as obtained from the LigParGen database [39, 40]. In this force field, some hydrogen atoms (of the TP molecule) have their twelfth order Lennard Jones (LJ) terms equal to 0, due to its “united atom” approach. Since this can possibly lead to artifacts in the geometry optimization, we appoint to those atoms the LJ parameters of hydrogen atom 2 in the TP molecule (Fig. 2), after reconfirming that this does not affect the accuracy.

With this force field, we optimize the geometry of ten randomly generated initial conformations using a trust-region-reflective algorithm [41, 42]. After this preoptimization, we attempt to search for the global minimum with a Genetic Algorithm (GA) [43, 44], using 10,000 individuals, and running for a maximum of 500 generations. Once the GA is finished, we refine its result with the same trust-region-reflective algorithm used before. This three-step optimization protocol is repeated five times before one final quantum chemical optimization with the GD3 B3LYP/cc-pVDZ level of theory. We then repeat this four-step optimization protocol again, independently, attaching from one up to three molecules of water.

Combining both a GA and trust-region-reflective algorithm allows us to overcome most of their respective limitations. While GAs have the advantage of being able to search for global minima, they do not (at least in our experiments) converge as quickly as the gradient-based methods.

Trust-region algorithms are widely used local minimizers that utilize the gradients and can be thought as improved versions of more standard line search methods. However, the trust-region-reflective algorithm slightly differs from the traditional ones, where instead of minimizing some arbitrary objective function $f: \mathbb {R}^{N} \longrightarrow \mathbb {R}$, through the following unconstrained problem:

$$\underset{x \in \mathbb{R}^{N}}{\arg\min} f \left( x \right) ,$$

a reflection transformation $R: \mathbb {R}^{N} \longrightarrow {\Omega }$, where ${\Omega } \subset \mathbb {R}^{N}$, is used to keep the *N* arguments of *f* (our optimization variables) within some arbitrary bounds, and minimizing instead the following unconstrained problem:

$$\underset{x \in \mathbb{R}^{N}}{\arg\min} f \left( R \left( x \right) \right) $$

This reflection transformation is modeled to mimic how an ideal mirror reflects a ray of light [41]. Like all local minimizers, this method is susceptible to get stuck in a local minimum which was clearly the case in our systems. In order to remedy this, an initially optimized initial guess from a global optimization method, such as a GA, can be used as the initial guess of the local minimizer.

GAs are a family of metaheuristic methods inspired by natural selection. In this family of methods, an arbitrarily sized set of individuals is created, where each of them will have a set of *N* chromosomes, if for example we aim to minimize some objective function $f : \mathbb {R}^{N} \longrightarrow \mathbb {R}$. These chromosomes consist of a string of “genes,” to encode a value of each optimization variable, per individual.

Often a binary basis is used, where each gene is either 0 or 1. However, other more complicated bases, such as a quaternary base [45], can be used. If a binary base is used, each chromosome has the values of an optimization variable encoded, for example, as a fixed point precision number. This means that in our case, each individual of our population will have six chromosomes for each molecule of water we attach to TP. Since we use internal coordinates, three of these represent the spacial translation of *x*, *y*, and *z* of the center of oxygen atom of a water molecule, while the remaining three represent the rotations of the water molecule.

In order to minimize our objective function, our population will undergo a selection process for some arbitrary number of generations (or iterations in more traditional terms). For this selection process, a fitness function is required (this can, for example, simply be the objective function *f* ). Each generation subset of the population with the best fitness values (if we were to use *f*, then the lowest values would be deemed best) are allowed to replicate, creating new individuals for the next generation, using the genetic material of both parents. Moreover, another subset, not necessarily equally sized to the set of individuals allowed to procreate, with the best fitness values, called the *elite*, are allowed to survive to the next generation.

**Fig. 3:**
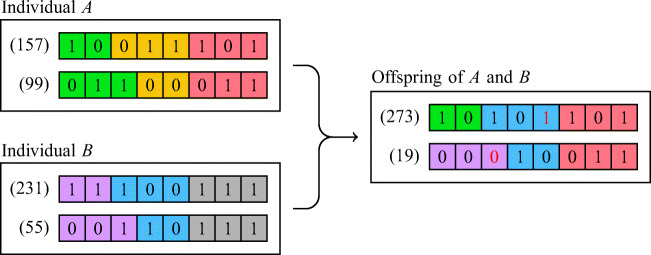
Genetic Algorithm: “Mating” between two individuals *A* and *B*. Each chromosome is divided into three arbitrarily sized strands, differentiated by the background color. Genes with numbers in red (in the offspring of *A* and *B*) have their values flipped due to mutations

In order for the individuals to replicate, at least two individuals are required to “mate.” The new offspring, created from this mating process, will have a new “genome” that is *mostly* a combination of the genome of its parents (see Fig. 3). This combination of chromosomes is carried over by a crossover function, by randomly combining the genetic material of the parents. Additionally, random “mutations” may occur by flipping the values of random genes of an offspring individual. In Fig. 3, an example of crossover and mutations of an offspring between two individuals *A* and *B* can be seen. Since each of these individuals has two chromosomes, they encode the two optimization variables of some objective function with two variables, each of them encoded using 8-bit integer precision, since each chromosome only has 8 genes (Fig. 3).

That said, if we allow at the very least the best individual of each generation to survive, the result of a GA will *necessarily* be equal or better the more generations we allow to pass. This means that, in our case, the best individual of the current generation will encode some internal coordinates for the molecules of water we want to attach to TP, with equal or smaller interaction energy, using our LJ model, compared to the internal coordinates encoded by the best individual of its previous generation.

In order to improve the convergence of this procedure, ten locally optimized values were supplied to the GA for its initial population, using MATLAB’s trust-region-reflective algorithm. This local algorithm required us to provide an exact implementation of the gradient of the interaction energies. Moreover, additional initial values were created by also using all the possible permutations by simply swapping the labels of the molecules of water, given that we treat them indistinguishably. These initial values are then used by a subset of our initial population to set the values of their chromosomes. The remainder of the population was randomly initialized by MATLAB’s GA default settings.

This global optimization routine was repeated (independently) ten times, attaching from one up to three molecules of water. The best result, for each case, was then used to calculate the Cartesian coordinates of all the atoms, to be later used in one final local optimization step by a quantum chemical calculation. The computer code Gaussian 16 [46] was used for all quantum chemical calculations, and our own MATLAB [47] based codes for the force-field–based optimizations and data analysis.

After studying the electronic structure of TP, we investigated the stability of various bonds that might be susceptible to dissociation. With the G4MP2 [48, 49] and CBS-QB3 [50, 51] chemistry models, we obtained dissociation thresholds of the bonds 7, 12, and 14. In a similar way, the proton affinity of oxygen atoms 13 and 17 were calculated for an isolate TP molecule. Differences in electronic densities and in partial charges [52], between anionic and neutral systems, with and without water were analyzed, along with the differences in the length of these bonds.

In addition to the trust-region-reflective algorithm and Genetic Algorithm, we also considered different combinations of methods using more traditional ones such as Interior-Point Algorithms (built-in in MATLAB’s Optimization Toolbox) and Simulated Annealing and Particle Swarm global global optimization methods (also built-in in MATLAB’s Global Optimization Toolbox). However, in our results, the combination of methods we described in more detail was overall the best one.

## Results and discussion

### Electron affinities

The experimental Adiabatic Electron Affinity (AEA) of O_2_ is 0.45 eV [53, 54], and all density functionals used were able to reproduce this within the margin of error. Experimental values of the Vertical Electron Affinity (VEA), of neither TP nor H_2_O, could be found. Theoretical values predicted by Arthur-Baidoo et al., for the AEA and VEA of TP, were 1.57 and 1.28 eV respectively (B3LYP/aug-cc-pVDZ), similar to the values we obtained with all functionals used in our calculations.

**Fig. 4:**
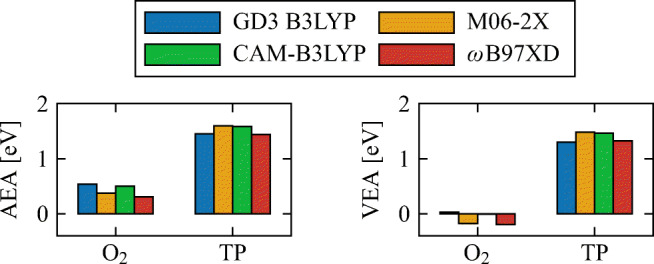
Adiabatic and vertical electron affinities of TP and O_2_ in gas phase, using different functionals with the basis set aug-cc-pVTZ

The AEA of both molecules was calculated as the difference of the energy of the anion and neutral molecules, both of them with their geometries optimized, while the VEA as the difference of the anion and neutral molecules both at the equilibrium geometry of the neutral system. The geometry optimization of TP both in its neutral and anion states was performed with the GD3 B3LYP functional and the aug-cc-pVDZ basis set, whereas for O_2_ the methods specified in Fig. 4 were used. All values shown for TP in the same figure are from single-point calculations using geometries previously optimized with the aug-cc-pVDZ basis set as well.

Both the AEA and VEA of TP have similar values with all the different functionals used, at about 1.5 eV. On the other hand, the AEA of O_2_ ranges between 0.3 and 0.5 eV, while its VEA was close to 0 eV. We note that from the AEA and VEA values, it can already be assumed that the anion is stable against dissociative electron transfer processes. This is further investigated below when bond dissociation energies are discussed.

### Electron distribution of the anion

The geometry optimization of TP and the surrounding water molecules was performed using the global optimization routine detailed in Section “Methods.” The results of this global optimization routine were used as initial values in more accurate quantum mechanical calculations, for both neutral and anion systems, which also involved another local optimization of the geometry. For this final geometry optimization, the functional and basis set GD3 B3LYP/aug-cc-pVDZ were used. All other quantum mechanical calculations using the larger basis set aug-cc-pVTZ were simply single-point energy calculations.

**Fig. 5:**
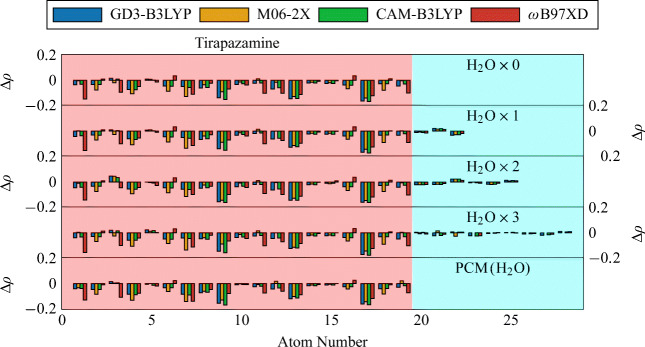
Mulliken charge differences between the anion and neutral systems of an isolated or hydrated TP molecule, using the basis-set aug-cc-pVTZ with different functionals. Both neutral and anionic systems are at the optimized geometry of the neutral system

**Fig. 6:**
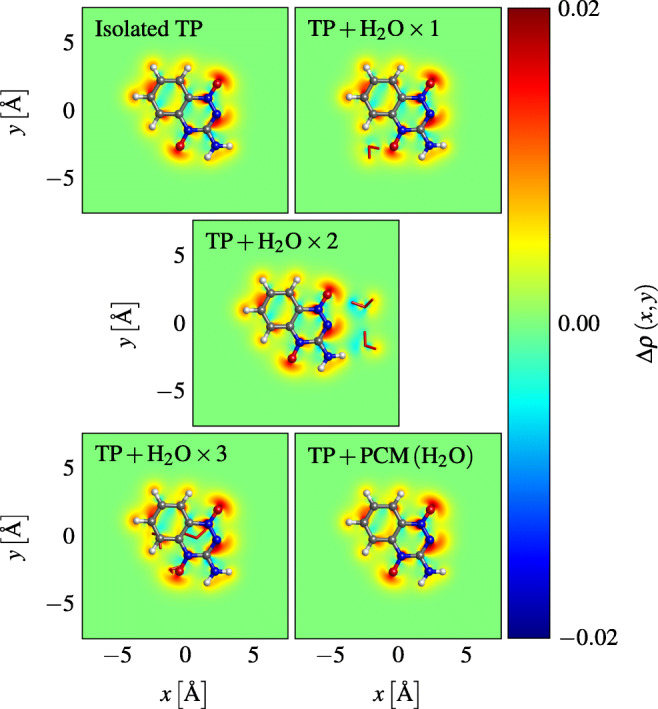
Excess electron density of an anion TP in isolated and hydrated environments, from GD3 B3LYP/aug-cc-pVDZ calculations

The Mulliken charge differences show that the extra electron resides mostly on hetero atoms 9, 13, and 17 (Fig. 5). This is consistent between the different density functionals, and is independent of how many molecules of water are attached to TP. The atoms in the molecules of water show little changes in their population whether attached to neutral or anionic TP. It can also be seen that micro-hydration as well as the water continuum had little influence on the resulting Δ*ρ* values. That is, the difference patterns remained very similar in all the atoms of TP, regardless of how many molecules of water we add in its vicinity. This is also seen in the electronic difference density maps of Fig. 6.

Atomic partial charges and charge differences have the advantage that they are scalar quantities and can be easily tabulated and compared with each other. They are, however, not physical observables, and depend on arbitrary assignments of the electron density to atoms. Since TP is a planar molecule, we display 2D maps of the electron densities by integrating along a perpendicular axis with respect to the molecular plane. We use these 2D maps to overcome the issues mentioned before. The corresponding maps are illustrated in Fig. 6. We define the excess density shown in Fig. 6 as:

$${\Delta} \rho \left( x, y \right) = {\int}_{- \infty}^{+ \infty} \rho^{A^{-}} \left( x, y, z \right) - \rho^{A} \left( x, y, z \right) \mathrm{d} z ,$$

where *A* is either an isolated or hydrated TP molecule, with some arbitrary number of water molecules (or solvated in a polarizable continuum).

**Fig. 7:**
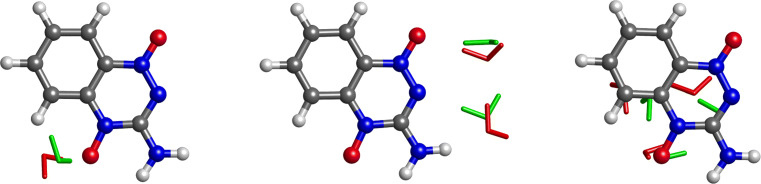
A TP molecule with one, two, and three molecules of water attached. Water molecules colored in red are in the conformation of the neutral system, and the ones colored in green in the conformation of the anionic system

The conformations of TP with one, two, and three molecules of water attached are depicted in Fig. 7. They were also derived by applying the optimization protocol described in Section “Methods.” The optimized geometry obtained from our global method was used as the initial guess not only for the neutral system but also for the anion system. The water molecules attached to neutral TP are marked in red, and the ones attached to anionic TP in green. Water favorably hydrogen-bonded at both oxygen atoms, and at nitrogen atom 9.

**Fig. 8:**
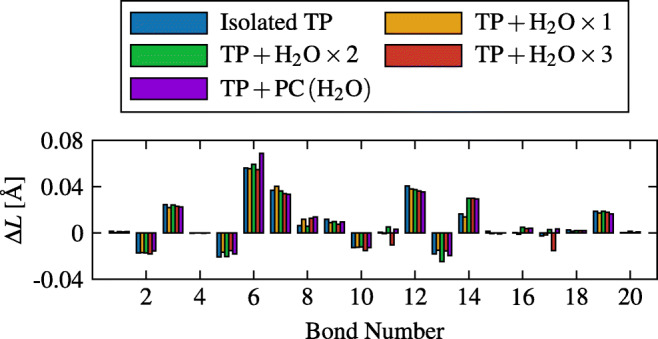
Changes in bond lengths in isolated and hydrated TP, upon attachment of an electron (GD3 B3LYP/aug-cc-pVDZ calculations)

Differences in the bond lengths (Δ*L*), between the atoms of TP, are small upon hydration (< 0.01 Å). No significant geometrical changes between TP in vacuo nor hydrated are observed either. On the other hand, anion formation leads to a small elongation of bonds 6, 7, and 12 roughly by 0.04 to 0.06 Å (Fig. 8). Twenty bonds are formed in a TP molecule. These are labeled from 1 to 20, as shown in the rightmost sketch of Fig. 2. Both the anion and neutral molecules remained with the same bonds in between atoms, with mostly negligible differences in the internal angles of these bonds.

Similarly to the Mulliken charge and the electronic density differences, between the anion and neutral systems, the differences between the bond lengths, also between the anion and neutral systems, remained almost unaltered upon microhydration or in a polarizable continuum of water. That is, the structural and electronic properties of TP seem to remain almost the same as in its isolated case, when exposed to an aqueous or biological environment.

### TP-Water interaction

**Fig. 9:**
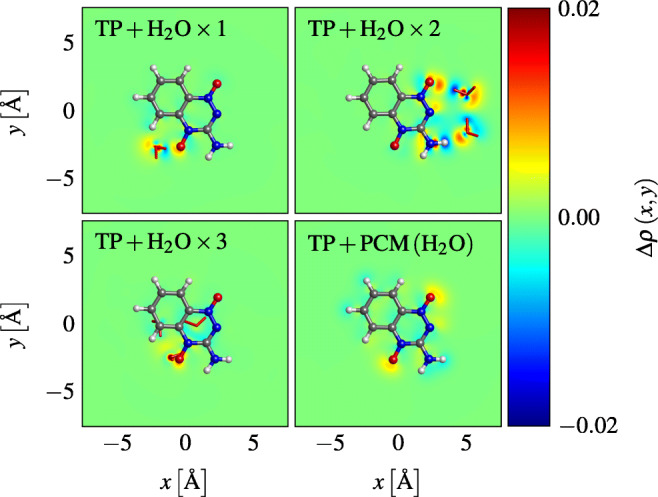
Changes of the electron density upon electron attachment to hydrated TP (GD3 B3LYP/aug-cc-pVDZ calculations)

Analogous to the 2D maps of Fig. 6 that show the excess density, Fig. 9 shows the changes in electron density when a hydrated TP molecule captures an electron. These changes are calculated through the following integral:

$$ \begin{array}{@{}rcl@{}} {\varDelta} \rho \left( x, y \right) &=& {\int}_{- \infty}^{+ \infty} \rho^{\text{TP} + \text{H}_{2}\text{O} \times N} \left( x, y, z \right) \\ &&- \rho^{\text{TP} } \left( x, y, z \right) - \rho^{\text{H}_{2}\text{O} \times N} \left( x, y, z \right) \mathrm{d} z, \end{array} $$

where TP and the *N* molecules of water are kept at the same internal coordinates as in the unseparated system with the density $\rho ^{\text {TP} + \text {H}_{2}\text {O} \times N}$.

With one molecule of water attached, a hydrogen bond between H_2_O and atom 13 of TP, as shown in Fig. 7, is formed in both neutral and anionic systems. However, by attaching two molecules of water, a hydrogen bond by oxygen 17 is formed instead, both in the neutral and anion systems, while the other H_2_O forms an oxygen bond with the hydrogen atom 15 of TP, in its neutral form. In the anionic system, however, the latter bond is replaced with a hydrogen bond with nitrogen atom 9 of TP. Attaching three molecules of water, a more abrupt change of the hydration patterns is observed. One of the molecules of water forms a hydrogen bond with the oxygen atom 13 of TP, while the remaining molecules of water form hydrogen bonds between each other. This pattern is observed both in the neutral and anion systems.

From Fig. 9, one sees that H_2_O causes a polarization of the heteroatoms of TP to which they are bonded, but nearly no charge transfer between TP and any of its surrounding molecules of water occurs. This is also in agreement with our previous results, since the surrounding water molecules affect the electronic properties of TP only mildly. This in turn can be understood by the high electron affinity of TP which causes the excess charge to be distributed exclusively among its atoms. As one would expect, the density shift is most prominent where TP interacts with water molecules, especially in the case of three water molecules.

**Fig. 10:**
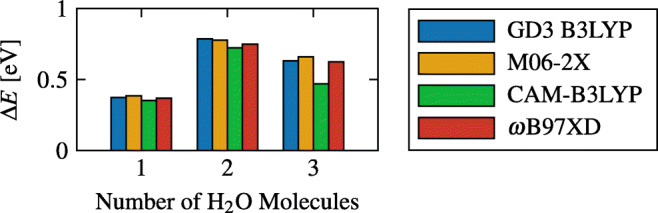
Binding energy of one water molecule as well as water dimer and trimer, to TP from different density functionals, using the basis set aug-cc-pVTZ

The binding energy between the molecules of water and TP, shown in Fig. 10, illustrates the following difference:

$$ {\varDelta} E = E \left( \text{TP} \right) + E \left( \text{H}_{2}\text{O} \times N \right) - E \left( \text{TP} + \text{H}_{2}\text{O} \times N \right), $$

where the *N* molecules of water, separated from TP, were kept at the exact same position and orientation they have when TP is not removed (as is also the case in Fig. 9).

The observed interaction between TP and a water trimer was lower in energy compared to the interaction between TP and a water dimer. From Fig. 7, we see that in the trimer water case, water-water bonds are mostly preferred, with only one TP-water bond being formed. However, in the dimer case, two TP-water bonds are formed. This unexpected behavior can be seen from the 2D maps in Fig. 9, where the 2D map of TP and the water trimer is mostly negligible (close to 0) in most places.

**Fig. 11:**
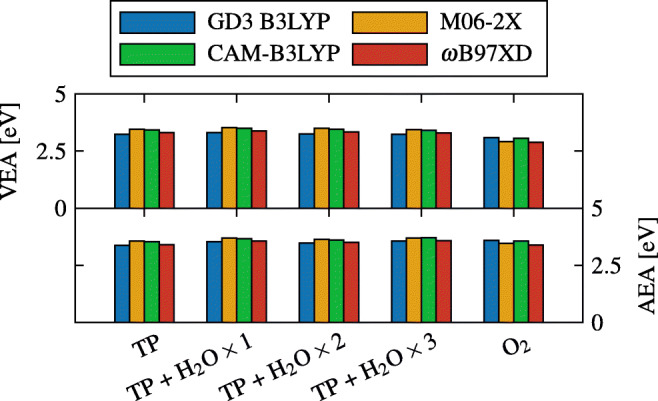
Electron affinities of TP from zero to three molecules of water attached, and O_2_ in a PCM of water, using different functionals with the basis set aug-cc-pVTZ

Microsolvating TP with water did not significantly alter neither its VEA nor its AEA when solvated in a PCM simulating water (Fig. 11). However, in vacuum conditions, a slight increase in the electron affinities is observed (both vertical and adiabatic), specially when the first molecule of water is added. A striking increase of the electron affinities of O_2_ is observed when solvated in water (using a PCM). In fact, in vacuum, O_2_ has significantly smaller electron affinities compared to TP; however, when solvated in water, its electron affinities nearly match TP’s electron affinities. Both the VEA and AEA, in vacuum, can be seen in Figs. S2 and S3, respectively, in the Supplementary Information.

The increase in the electron affinities of O_2_ (both vertical and adiabatic) when solvated in water is still not enough to explain the remarkable hypoxic radiosensitization selectivity of TP, since both electron affinities are nearly identical to their respective counterparts of TP.

### Bond dissociation energies

As shown in Fig. 4, the electron affinity of TP is relatively high. This often indicates a very stable anion, although it is in principle possible for a dissociation channel to open up. Bond dissociation (BD) in TP is intrinsically related to its biological activity as a radiosensitizer.

We have shown that microhydration of TP did neither noticeably change its electron affinity nor the charge distribution of its anion. Therefore, we can restrict our discussion of BD processes to TP in gas phase and in a PCM of water. Several BD reactions were calculated using the quantum thermochemical extrapolation methods G4MP2 and CBS-QB3, which are designed to deliver accurate free energies for such reactions. Both methods are independent of each other, and in all cases the energies are in good agreement. Most dissociation thresholds of TP in gas phase are around 2.5 to 3.0 eV; however, when solvated in a PCM of water, cleavage of an O^−^ from a TP anion is substantially lower compared to gas phase (Fig. 13). Nonetheless, all dissociation reactions remained endothermic.

Electron transfer between TP and O_2_, as well as the cleavage of the bonds of either of the two oxygen atoms in TP, in its neutral or anion state, is endothermic by more than 1.2 eV. Consequently, these processes are unlikely to play a major role in explaining TP’s unique selective radiosensitizing properties. However, most experimental studies we reviewed, to this day, which used TP either in vitro or in vivo were also accompanied with ionizing radiation. The by-products of radiolysis of water molecules, namely hydrogen radicals, could play a role in facilitating the cleavage of these bonds.

Despite detachment of a  radical from a protonated TP being very exothermic in our calculations (> − 14 eV), it alone does not explain TP’s selective behavior. Protonation is more likely to occur in acidic environments. However, despite pH levels varying very noticeably between different organelles of human cells, for example about 4.5 in the lysosome and about 8.0 in the mitochondria [55], tumorous cells prefer alkaline intracellular pH levels [56, 57] (Table 1).

**Table 1:** Thresholds of formation of HO_2_ using different methods, both in vacuum and in a PCM of water

	Vacuum		PCM(H_2_O)	
	G4MP2	CBS-QB3	G4MP2	CBS-QB3
${\varDelta } E \left (\text {O}_{2}^{\hphantom {-}} + \text {H} \longrightarrow \text {HO}_{2}\right )$	− 2.15	− 2.11	− 2.15	− 2.26
${\varDelta } E \left (\text {O}_{2}^{-} + \text {H} \longrightarrow \text {HO}_{2}^{-} \right )$	− 2.82	− 2.73	− 3.01	− 2.93

All energies are expressed in eV

**Fig. 12:**
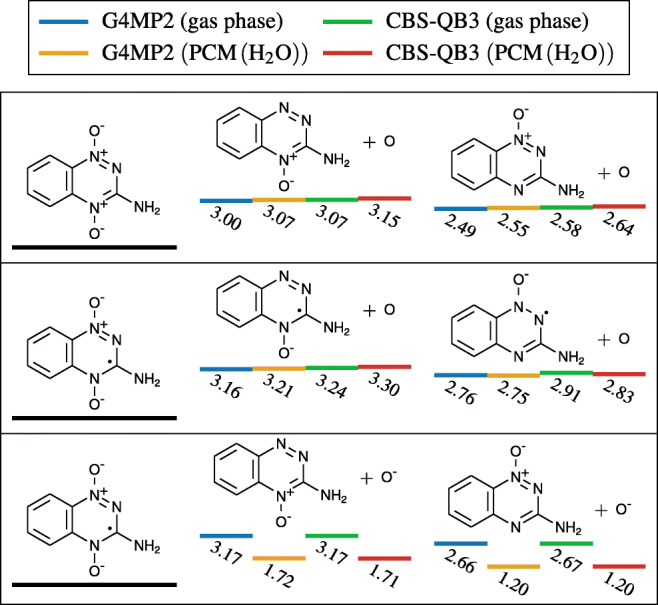
Energy thresholds of several chemical reactions capable of producing DNA damaging ROS, via hydrogen capture of a neutral (left) and anion (right) TP using different methods. All energies are expressed in eV. Bottom right values in a PCM of water failed to converge with either of the two used methods

Capture of a hydrogen radical followed by detachment of  from a TP anion is exothermic by − 1.52 to − 1.19 eV, as depicted in Fig. 12. These reactions are likely to become more favorable on irradiated tissue, where radiolysis of water molecules can occur. Recalling that hydrogen radicals are one of the by-products of this reaction and TP’s high reliance on hydrogen radicals to produce hydroxyl radicals (through these pathways) could possibly serve to explain TP’s selective radiosensitizing properties.

**Fig. 13:**
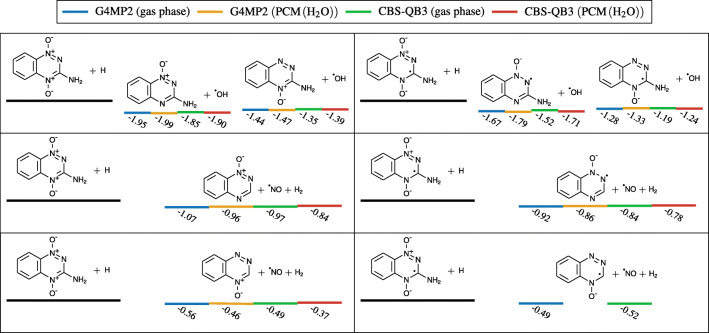
Bond dissociation thresholds of both oxygen atoms, in neutral (top) and anionic (mid and bottom) TP molecules, using different methods. All energies are expressed in eV

This hypothesis is supported by the fact that formation of HO_2_ radical via hydrogenation of an O_2_ molecule is exothermic by at least − 2.15 eV, without a reaction barrier, both in vacuum and solvated (PCM) in our calculations. Therefore, normoxic conditions should make all mechanisms proposed in Fig. 12 less likely to occur, thus providing a possible explanation for TP’s selective hypoxic radiosensitization. This argument can also be extended to the formation of $\text {HO}_{2}^{-}$ by subsequent electron capture (Fig. 13).

**Fig. 14:**
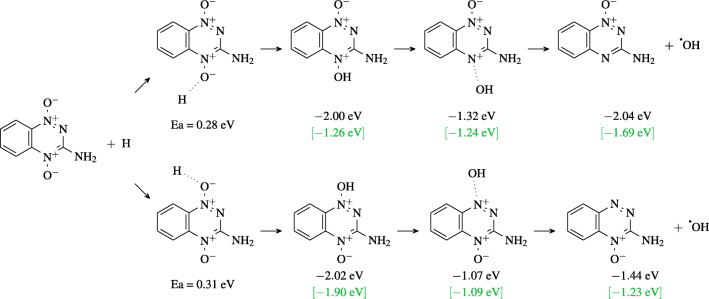
Energy profiles of OH radical formation reactions, differing by which oxygen atom of TP ends up in OH. For both cases, the energies refer to neutral TP (energies in black) and TP anion (energies in green brackets) as reactant. M06-2X/6-31G(d,p) calculations

All reactions shown in Fig. 12 are likely to involve multiple reaction steps. This is especially obvious for the ones producing NO fragments. The thermochemical extrapolation methods which were used for the reaction free energies are not applicable here. Instead, we obtained the reaction profiles from M06-2X/6-31G(d,p) calculations. The M06-2X functional has been briefly discussed in Section “Calculations” and is regarded to be reliable for transition state energies. We resorted to the 6-31G(d,p) basis set because the large number of calculations necessary for identifying the reaction pathway would be cumbersome with the aug-cc-pVTZ basis used mostly in this work. In the reactions where OH is formed, the first step is the protonation of one of the two O atoms, followed by breaking of one N-O bond to produce an OH radical and benzotriazinyl. In the neutral system, the barriers of this step are about 0.3 eV for both oxygens. The second step, the N-O bond dissociation, is rate determining. The dissociation of the O atom neighboring the NH_2_ group has a lower activation barrier than the other one (0.68 vs. 0.95 eV). For the anionic system, the same pathway is preferred (0.02 vs. 0.81 eV). Thus, for the OH formation reaction, thermodynamic and kinetic control leads to the same conclusions. Diagrams of the reactions are shown in Fig. 14.

Finally, we investigated the energetic conditions of dissociation of  radicals from TP. Nitric oxide radicals have been shown to be as efficient as O_2_ at enhancing cell death in vitro [58]. These reactions might occur if energy is provided by another partner. In Fig. 12, this is shown for a H radical reacting with TP, a reaction which is equivalent to the formation of TP anion and subsequent protonation. In these reactions, the formation of  radicals is exothermic by at least 0.5 eV.

The formation of NO starts similar to the formation of OH, but requires a much more complicated rearrangement. It consists of the move of one O atom to the carbon where the NH_2_ group resides (carbon number 7 in Fig. 2), the subsequent bonding of this O atom to N of NH_2_, and the dissociation of NO. It involves several “small” steps: (1) protonation of O, (2) N-O bond breaking to form OH radical, (3) OH attacking, (4) dehydrogenation, (5) C-O bond formation, (6) proton shifting and N-O bond breaking to form NO radical. The profile of these reactions is shown in Fig. S1 of the Supplementary Information in a similar way to Fig. 14 for neutral and anionic systems, and the two possible oxygens involved. In all cases, high barriers are encountered when OH and NH_2_ start to mix, 5.5–6.0 eV for the neutral and 4.0–4.7 eV for the anionic case. We were not able to find a pathway with smaller barriers despite extensive searches and must at present conclude that NO formation would be quenched due to kinetic reasons.

## Conclusions

We find that TP forms a very stable anion and that various bond-breaking channels are highly endothermic. Hydration affects the energetics of TP only to a small degree. However, protonated or hydrogenated TP behaves differently, and several exothermic reaction channels can open up, leading to ROS formation.

The high electron affinity of TP should prevent any electron transfer between TP’s anion and a neighboring oxygen molecule, contrary to what is often suggested (see Fig. 1). The selectivity of TP must, therefore, be the result of a more elaborate process, other than direct generation of oxygen radical anions in healthy cells. One such possibility might be due to the formation of  radicals, when either of its two oxygen atoms captures a neighboring hydrogen atom, and subsequently get detached from the parent molecule (TP).

Based on our quantum mechanical calculations, we suggest that TP’s observed selectivity as a radiosensitizer can be explained by the following: (1) The lower GSH and GSSG ratios in tumorous cells make these cells less capable of fixing DNA damage, according to the OFH, from any ROS formed from a TP anion. (2) Radiolysis of the water molecules which increases the likelihood of TP capturing a hydrogen radical, which can then be followed by a detachment of a DNA damaging hydroxyl radical, or nitric oxide radical. (3) Formation of HO_2_ in normoxic cells which could inhibit TP to detach ROS fragments.

Moreover, our calculations could also explain the reported cytotoxicity potentiation by SR 4317 which was reported by Siim et al. when used alongside TP [20]. SR 4317 is almost identical to TP and both molecules can decompose by formation of a hydroxyl radical.

## Electronic supplementary material

Below is the link to the electronic supplementary material.

(DOCX 220 KB)
