# Supplementary material for: Electronic structure and reactivity of tirapazamine as a radiosensitizer
Source: J Mol Model. 2021 May 22;27(6):177. doi: 10.1007/s00894-021-04771-8 (PMC8140980; doi:10.1007/s00894-021-04771-8)
Supplement: Supplementary file 1 — (DOCX 220 KB) [file 894_2021_4771_MOESM1_ESM.docx]

**Chemical reactions capable of producing DNA damaging ROS**

| 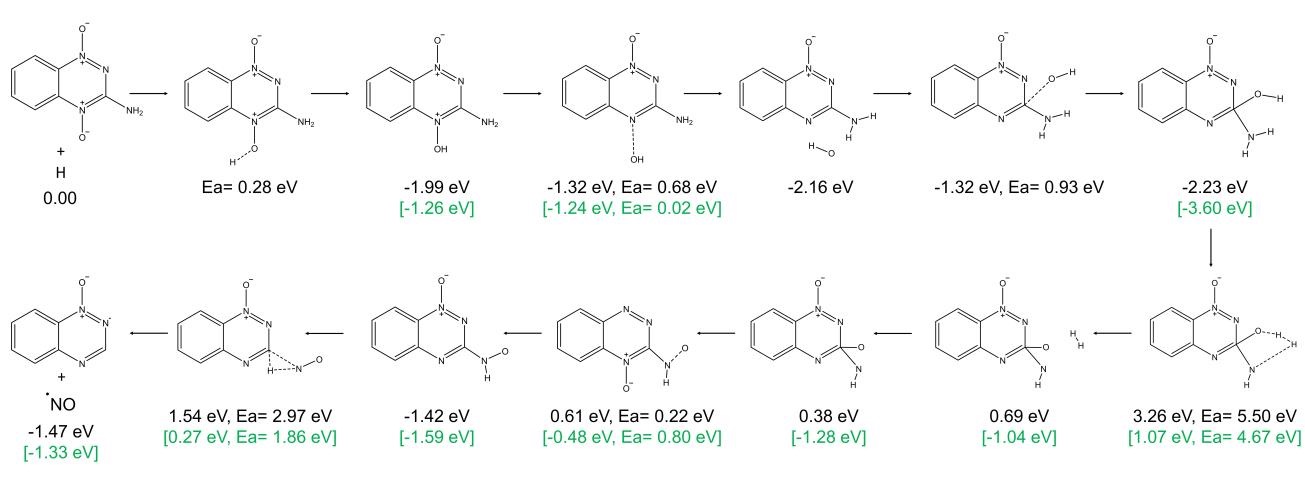 |
| --- |
| 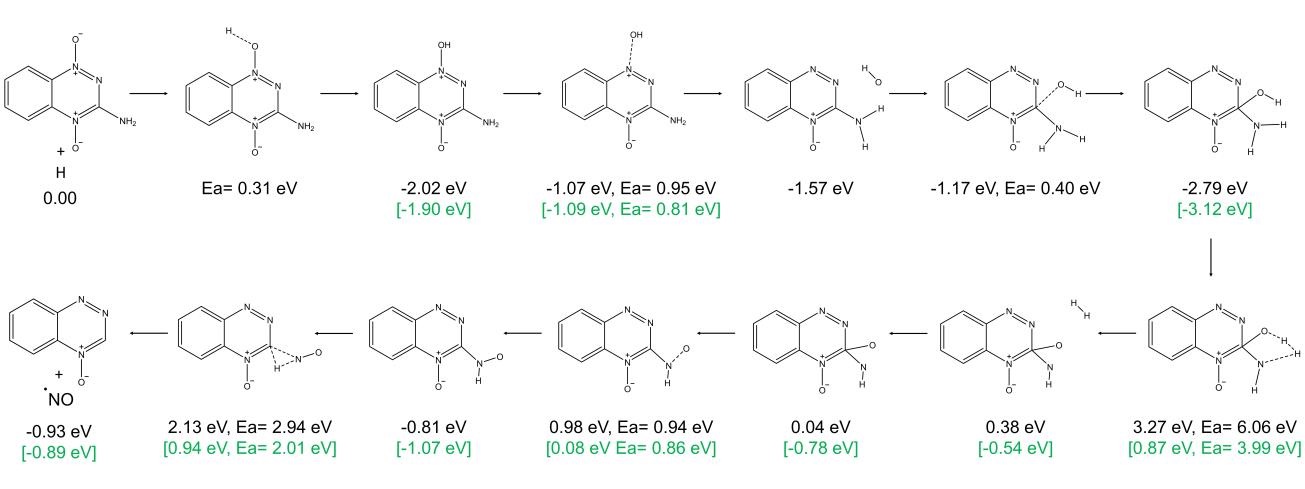 |

Figure S1 Energy profile of the NO radical formation reaction. top: starting from neutral TP (energies in black) and starting from TP anion (energies in green), from M06-2X/6-31G(d,p) calculations.

**Electron Affinities (PCM(H_2_O))**

Table S1 Vertical electron affinities of TP from zero to three molecules of water attached, andO_2_ solvated in a PCM simulating water, using different functionals with the basis-set aug-cc-pVTZ.

|  | GD3 B3LYP | CAM-B3LYP | M06-2X | wB97XD |
| --- | --- | --- | --- | --- |
|  | aug-cc-pVTZ | aug-cc-pVTZ | aug-cc-pVTZ | aug-cc-pVTZ |
|  | [eV] | [eV] | [eV] | [eV] |
| VEA[TP] | 3,23 | 3,42 | 3,46 | 3,30 |
| VEA[TP + H_2_O x 1] | 3,31 | 3,50 | 3,53 | 3,38 |
| VEA[TP + H_2_O x 2] | 3,25 | 3,45 | 3,50 | 3,34 |
| VEA[TP + H_2_O x 3] | 3,23 | 3,41 | 3,45 | 3,29 |
| VEA[O_2_] | 3,09 | 3,05 | 2,92 | 2,88 |

Table S2 Adiabatic electron affinities of TP from zero to three molecules of water attached, and O_2_, solvated in a PCM simulating water, using different functionals with the basis-set aug-cc-pVTZ.

|  | GD3 B3LYP | CAM-B3LYP | M06-2X | wB97XD |
| --- | --- | --- | --- | --- |
|  | aug-cc-pVTZ | aug-cc-pVTZ | aug-cc-pVTZ | aug-cc-pVTZ |
|  | [eV] | [eV] | [eV] | [eV] |
| AEA[TP] | 3,38 | 3,53 | 3,56 | 3,41 |
| AEA[TP + H_2_O x 1] | 3,54 | 3,66 | 3,70 | 3,56 |
| AEA[TP + H_2_O x 2] | 3,48 | 3,61 | 3,64 | 3,50 |
| AEA[TP + H_2_O x 3] | 3,57 | 3,72 | 3,70 | 3,58 |
| AEA[O_2_] | 3,60 | 3,57 | 3,47 | 3,39 |

**Electron Affinities (gas phase)**

Table 3 Vertical electron affinities of TP from zero to three molecules of water attached, and O_2,_ in gas phase, using different functionals with the basis-set aug-cc-pVTZ.

|  | GD3 B3LYP | CAM-B3LYP | M06-2X | wB97XD |
| --- | --- | --- | --- | --- |
|  | aug-cc-pVTZ | aug-cc-pVTZ | aug-cc-pVTZ | aug-cc-pVTZ |
|  | [eV] | [eV] | [eV] | [eV] |
| VEA[TP] | 1,30 | 1,47 | 1,48 | 1,33 |
| VEA[TP + H_2_O x 1] | 1,46 | 1,63 | 1,64 | 1,49 |
| VEA[TP + H_2_O x 2] | 1,42 | 1,59 | 1,61 | 1,45 |
| VEA[TP + H_2_O x 3] | 1,32 | 1,48 | 1,49 | 1,34 |
| VEA[O_2_] | 0,03 | -0,01 | -0,18 | -0,20 |

Table 4 Adiabatic electron affinities of TP from zero to three molecules of water attached, and O2, in gas phase, using different functionals with the basis-set aug-cc-pVTZ.

|  | GD3 B3LYP | CAM-B3LYP | M06-2X | wB97XD |
| --- | --- | --- | --- | --- |
|  | aug-cc-pVTZ | aug-cc-pVTZ | aug-cc-pVTZ | aug-cc-pVTZ |
|  | [eV] | [eV] | [eV] | [eV] |
| AEA[TP] | 1,45 | 1,59 | 1,60 | 1,44 |
| AEA[TP + H_2_O x 1] | 1,78 | 1,89 | 1,91 | 1,77 |
| AEA[TP + H_2_O x 2] | 1,79 | 1,90 | 1,91 | 1,78 |
| AEA[TP + H_2_O x 3] | 1,85 | 1,99 | 1,97 | 1,85 |
| AEA[O_2_] | 0,54 | 0,50 | 0,37 | 0,31 |

Figure S2 Vertical electron affinities of TP from zero to three molecules of water attached, and O2, in vacuum, using different functionals with the basis-set aug-cc-pVTZ.

Figure S3 Adiabatic electron affinities of TP from zero to three molecules of water attached, and O2, in vacuum, using different functionals with the basis-set aug-cc-pVTZ.
